# Engineering Human T Cells for Resistance to Methotrexate and Mycophenolate Mofetil as an *In Vivo* Cell Selection Strategy

**DOI:** 10.1371/journal.pone.0065519

**Published:** 2013-06-06

**Authors:** Mahesh Jonnalagadda, Christine E. Brown, Wen-Chung Chang, Julie R. Ostberg, Stephen J. Forman, Michael C. Jensen

**Affiliations:** 1 Departments of Cancer Immunotherapeutics & Tumor Immunology, and Hematology and Hematopoietic Cell Transplantation, Beckman Research Institute, City of Hope, Duarte, California, United States of America; 2 Ben Towne Center for Childhood Cancer Research, Seattle Children’s Research Institute, Seattle, Washington, United States of America; University of Minnesota, United States of America

## Abstract

Gene transfer and drug selection systems that enforce ongoing transgene expression *in vitro* and *in vivo* which are compatible with human pharmaceutical drugs are currently underdeveloped. Here, we report on the utility of incorporating human enzyme muteins that confer resistance to the lymphotoxic/immunosuppressive drugs methotrexate (MTX) and mycophenolate mofetil (MMF) in a multicistronic lentiviral vector for *in vivo* T lymphocyte selection. We found that co-expression of human dihydrofolate reductase (DHFR^FS^; L22F, F31S) and inosine monophosphate dehydrogenase II (IMPDH2^IY^; T333I, S351Y) conferred T cell resistance to the cytocidal and anti-proliferative effects of these drugs at concentrations that can be achieved clinically (up to 0.1 µM MTX and 1.0 µM MPA). Furthermore, using a immunodeficient mouse model that supports the engraftment of central memory derived human T cells, *in vivo* selection studies demonstrate that huEGFRt^+^DHFR^FS+^IMPDH2^IY+^ T cells could be enriched following adoptive transfer either by systemic administration of MTX alone (4.4 -fold), MMF alone (2.9-fold), or combined MTX and MMF (4.9-fold). These findings demonstrate the utility of both DHFR^FS^/MTX and IMPDH2^IY^/MMF for *in vivo* selection of lentivirally transduced human T cells. Vectors incorporating these muteins in combination with other therapeutic transgenes may facilitate the selective engraftment of therapeutically active cells in recipients.

## Introduction

A continuing unmet need for genetically engineered cellular therapies is the development of drug selection platforms that are non-immunogenic, and, that enable selection to occur either *in vitro* or *in vivo* in humans. While a number of drug-resistance enzymes have been employed for selection of gene modified cells, including O^6^-mehtylguanine-DNA-methyltransferease (MGMT), multidrug resistance associated protein 1 (MDR1), bacterial hygromycin resistance gene (Hy) and neomycin phosphotransferase (*neo*) variants [1], many of these selective transgenes have proven disadvantageous in the clinic. For example, transgenes of non-human origin used for *in vitro* selection (e.g., Hy, *neo*, and Herpes simplex thymidine kinase, HSV-tk), often lead to immunological rejection of gene-modified cells [2]–[6]. Additionally, when MDR1-transduced autologous CD34^+^ cells were transferred into cancer patients, no enrichment of transduced cells was detected following etoposide treatment [7], [8]. Previous clinical trials of MGMT-, *neoR-* and Hy- mediated selection have also been halted due to safety concerns with long-term administration of selection drugs, (i.e., with DNA-alkalizing agents, neomycin, and hygromycin respectively) [1], [9]. Thus, there is a need for alternative strategies that will enable drug selection of gene modified cells with a tolerable toxicity profile in human patients.

Genetically engineered T cells expressing scFv chimeric receptors or TCR transgenes hold significant promise for the treatment of infectious and malignant diseases [10]–[14]. The therapeutic responses have been shown to correlate with the levels of long-term T cell persistence following adoptive transfer of gene-engineered T cells to patients [10]. While depletion of lymphocytes and exogenous cytokine administration can improve T cell persistence, their effects are not uniform [15]. One potential approach to further improve T cell persistence is to develop more effective *in vivo* selection strategies for gene-engineered cells in humans. One strategy would be the inclusion of a drug-resistance gene that would provide a selective proliferative advantage to the gene-modified cells upon drug administration to patients.

Two drugs of potential utility in such a strategy are methotrexate (MTX) and mycophenolate mofetil (MMF), which competitively inhibit dihydrofolate reductase (DHFR), involved in synthesis of thymidylate nucleotides [16], and inosine-5′- monophosphate dehydrogenase II (IMPDH2), a rate-limiting enzyme in the *de novo* synthesis of guanosine nucleotides [17], [18] respectively. Proliferation of T and B cells is dependent on the activity of both DHFR and IMPDH2 [19], and thus MTX and MMF are known to inhibit the proliferation and survival of T lymphocytes [20]. Previous studies demonstrate that a double point mutation in the human IMPDH2 gene, substituting both Thr333 to Ile, and Ser351 to Tyr (IMPDH2^IY^) [8] confers resistance to mycophenolic acid (MPA), an active metabolite of MMF. Likewise, a double point mutant of human DHFR with substitutions of Leu22 to Phe, and Phe31 to Ser (DHFR^FS^) [16], confers resistance to MTX. The products of these two mutant transgenes decrease binding to MTX and MMF (prodrug of MPA) [21], while retaining enzymatic activity in synthesizing purine and pyramidine nucleotides [20]. Expression of the trans-dominant DHFR^FS^/IMPDH2^IY^ genes is therefore hypothesized to permit the *in vivo* selection of transduced cells with MTX/MMF without disabling nucleotide synthesis.

The objective of this study was to confer dual resistance of primary human T cells to MTX and MMF for the purpose of mediating selection of gene-modified T cells *in vivo* when treated with either drug alone or both drugs. Here, we investigated the ability of DHFR^FS^ and IMPDH2^IY^ to confer resistance of primary human T cells to MTX and MMF both *in vitro,* and in an *in vivo* mouse xenograft model. Overall, we found that the expression of DHFR^FS^ and IMPDH2^IY^ supported the preferential expansion and selection of transduced over non-transduced T cells following administration of MTX and MMF at dosing schedules that were minimally toxic to animals.

## Results

### Gene Modification of Human Central Memory Derived T cells for MTX and MMF Resistance

**Figure 1:**
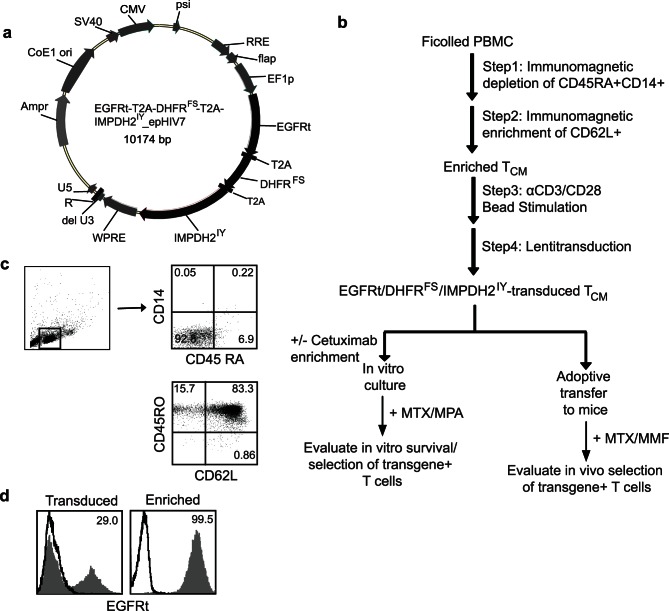
Experimental system for evaluating selection of gene-modified T cells in NSG mice. (**a**), Plasmid map of construct containing drug resistance genes DHFR^FS^ and IMPDH2^IY^, huEGFRt and T2A gene sequences (black) that was used to genetically alter primary human T cells. Lentiviral vector backbone (epHIV7) - related sequences are depicted in grey. (**b**), Schematic for isolation, genetic modification and selection of primary human T cells. (**c**), CD45RA and CD14 staining of mononuclear cells after sorting from PBMC (top), and CD62L and CD45RO staining of T cells after enriching from PBMC (bottom). The percent positive cells in each quadrant are indicated. (**d**), Primary human T cells were transduced with above lentiviral vector; transduced T cells and immunomagnetic sorted EGFRt^+^ T cells were stained for EGFRt expression and analyzed for transduction efficiency by flow cytometry. The percent EGFRt^+^ T *(*grey histogram; isotype control-dark line) cells are depicted.

To compare MTX- and MMF-mediated cell selection strategies, either singly or in combination, we designed a lentiviral vector to direct the co-expression of DHFR^FS^, IMPDH2^IY^ and a truncated human EGF receptor (huEGFRt) [22]. The transgenes are expressed from a single EF-1α promoter, with each polypeptide sequence separated by the ribosomal skip T2A sequence [23] for translation of three proteins from one transcribed message (**Fig. 1a**). EGFRt functions as a way to mark gene modified cells and allows for alternative cell selection via Erbitux™ [22]. We chose to evaluate MTX and MMF drug selection in central memory derived T (T_CM_) cells, a sub-population of CD62L^+^CD45RO^+^ T cells, which have been shown to have favorable properties for therapeutic application including the capacity for self renewal, proliferation, long-term persistence, and an ability to differentiate into effector T cells [10], [15], [24]. As previously published, we consistently enrich T_CM_ cells to greater than 70% purity from peripheral blood by first depleting naïve T cells (CD45RA) and monocytes (CD14), then positively selecting for CD62L^+^ cells (**Fig. 1b and 1c**) [25]. Here, the enriched T_CM_ (83% CD62L^+^CD45RO^+^, **Fig. 1c**) were then transduced with an EGFRt-T2A-DHFR^FS^-T2A-IMPDH2^IY^_epHIV lentiviral vector (**Fig. 1a**), which typically yields greater than 20% cell transduction as assessed by cell surface expression of huEGFRt (**Fig. 1d**). These engineered cells could then be further immunomagnetically enriched to greater than 98% purity with cetuximab based on expression of huEGFRt (**Fig. 1d**).

### huEGFRt/DHFR^FS^/IMPDH2^IY^-engineered Primary Human T_CM_ are Resistant to Cytocidal Concentrations of MTX and MPA

**Figure 2:**
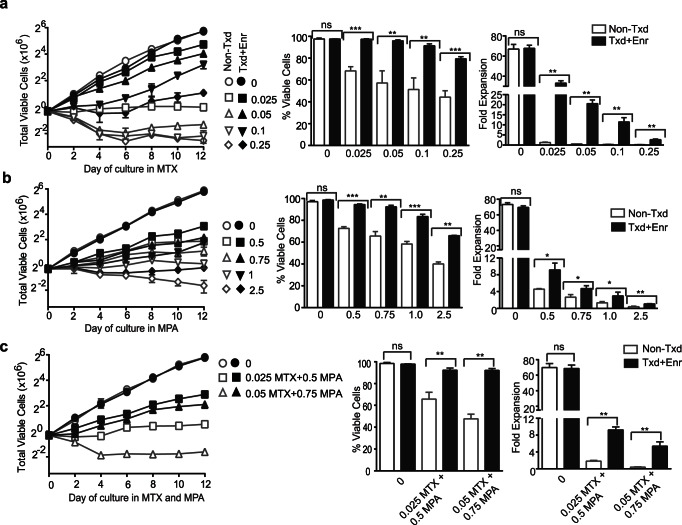
Primary human T cells transduced to DHFR^FS^/IMPDH2^IY^ transgenes are resistant to MTX and MPA. Non-transduced T cells (non-Txd; grey line/bar) and immunomagnetically-enriched EGFRt^+^ T cells (99.5% EGFRt^+^; Txd+Enr; black line/bar) were plated on day 8 at the indicated concentrations of MTX (**a**), MPA (**b**), and a combination of MTX+ MPA (**c**), cells were followed for total viable cell number, percentage of viable cells, and fold expansion for12 days. Equal numbers of cells were plated in triplicate wells of 24-well plates. The data represent the mean ± S.D. There was a significant difference in viability and fold expansion at day 12 between the non-transduced (Non-Txd) and the transduced, EGFRt-enriched T cells (Txd+Enr) at each drug concentration. ***, p≤0.0002; **, p≤0.001; *, p≤0.01. The data are representative of three separate experiments.

To examine the resistance of huEGFRt/DHFR^FS^/IMPDH2^IY^ **-**transduced T_CM_ to MTX and MPA, we first examined engineered cells that had been immunomagnetically enriched for huEGFRt^+^ expression (>98% purity; **Fig. 1d**). As expected, huEGFRt-enriched cells maintained a T cell phenotype as assessed by surface expression of CD4, CD8, CD28, CD45, TCRαβ and CD127 (**Fig. S1**), and displayed equally potent functional activity, as compared to non-transduced control T_CM_ (**Fig. S1**). These uniformly positive huEGFRt (>98%) -expressing populations enabled us to determine the optimal drug selection concentrations for huEGFRt^+^/DHFR^FS^/IMPDH2^IY^-engineered T_CM_. To this end, non-transduced vs. transduced and EGFRt-enriched (EGFRt^+^) cells were plated in MTX (0–0.25 µM) (**Fig. 2a**), MPA (0–2.5 µM) (**Fig. 2b**), and combinations of both (**Fig. 2c**). In the absence of MTX and MPA, the non-transduced and EGFRt^+^ T cells expanded at an equivalent rate (70.0±4.6 and 68.6±3.0 fold). Following incubation with MTX for 12 days, non-transduced T cells did not expand at concentrations ≥0.05 µM MTX and a decrease in viability from 92% to 57.1±11.2% (**Fig. 2a**). In contrast, huEGFRt^+^/DHFR^FS^/IMPDH2^IY^ T cells expanded 20.5±1.8-fold at 0.05 µM and 11.4±2.1-fold at 0.1 µM MTX with 93.6±2.7% viable cells. (**Fig. 2a**). In the presence of MPA, huEGFRt^+^/DHFR^FS^/IMPDH2^IY^ T cells expanded 4.7±0.6-fold at 0.75 µM and 3±0.9-fold at 1 µM MPA over 12 days with 83.4±2.1–92.3±1.4% viable cells, whereas non-transduced cells did not expand and cell viability decreased to 65.6±4.1% and 58.3±2.3% at these concentrations (**Fig. 2b**). Furthermore, we analyzed the survival and expansion of huEGFRt^+^/DHFR^FS^/IMPDH2^IY^ T cells in media containing combinations of 0.025 µM MTX +0.5 µM MPA and 0.05 µM MTX +0.75 µM MPA over 12 days. The transduced, huEGFRt-enriched T cells expanded 9.2±0.7 and 5.4±1.0-fold, respectively, with 92.2±1.6% viability, whereas non-transduced cells did not expand and cell viability decreased to 65.7±6.3% and 47.6±4.3% (**Fig. 2c**). These data indicate that both DHFR^FS^ and IMPDH2^IY^ can confer drug resistance to primary human T cells, allowing them to expand and maintain high cell viability (≥90%) upon culture in MTX and MPA. The growth mediated by DHFR^FS^ in the presence of MTX was more robust than that mediated by IMPDH2^IY^ in MPA over the short-term two-week culture period.

### huEGFRt/DHFR^FS^/IMPDH2^IY^ Primary Human T cells Maintain their Phenotype and Function Following Growth in MTX and MMF

**Figure 3:**
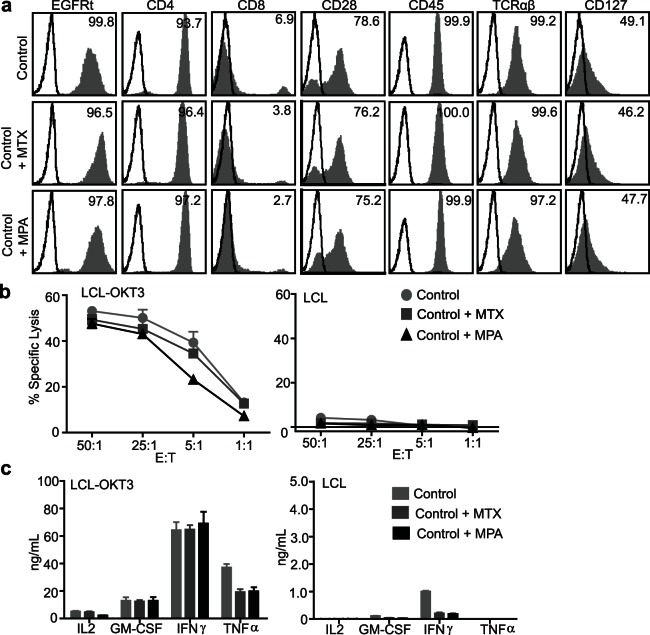
Immunomagnetically enriched huEGFRt/DHFR^FS^/IMPDH2^IY^ T cells maintained their cell surface phenotype and effector function upon culture in MTX and MPA. (**a**), Cell surface expression of EGFRt, CD4, CD8, CD28, CD45, TCRαβ and CD127 (grey histogram) vs. isotype control antibody (open histogram) on EGFRt-enriched T cells after 14 days culture +/−0.1 µM MTX or 1 µM MPA were analyzed by flow cytometry. Percentage of positive cells is indicated. (**b**), Cytotoxic activity of the same cells in (**a**) after 4 hr co-culture with ^51^Cr- labeled OKT3-expressing LCL (LCL-OKT3) or negative control LCL targets. Mean percent of ^51^Cr release ± S.D. of triplicate wells is depicted. (**c**), Production of cytokines IL-2, GM-CSF, IFN-γ and TNF-α by the same cells as described in (**a**). Supernatants were collected after overnight co-culture of the same cells in (a) with LCL-OKT3 or negative control LCL stimulators, and mean (±S.D. of triplicate wells) cytokine levels were quantified by cytometric bead array.

MTX and MPA are lymphotoxic drugs known for their cytocidal activity as well as effects to suppress lymphocyte proliferation [16]–[20]. We therefore assessed whether MTX or MPA, at drug concentrations optimized above for proliferation and viability, alter the phenotype or function of huEGFRt/DHFR^FS^/IMPDH2^IY^ T_CM_. For these studies, transgene expressing huEGFRt/DHFR^FS^/IMPDH2^IY^ T_CM_ was EGFRt-enriched and then treated with or without 0.1 µM MTX or 1 µM MPA for 14 days. The effect of DHFR^FS^ and IMPDH2^IY^ expression and MTX and MPA selection on skewing the phenotype and function of primary human T_CM_ is unknown. In the presence or absence of either MTX or MPA, huEGFRt/DHFR^FS^/IMPDH2^IY^ T_CM_ expressed comparable levels of CD4, CD8, CD28, CD45, TCRαβ and CD127 surface expression (**Fig. 3a**). Further, using lymphoblastoid cells that express the CD3 agonist OKT3 (LCL-OKT3) as stimulators, the cytolytic potency and activation-dependent cytokine production (IL-2, GM-CSF, IFN-γ and TNF-α) of enriched huEGFRt/DHFR^FS^/IMPDH2^IY^ T cells were identical in the presence or absence of either MTX or MPA (**Fig. 3b, c**). Together, these results suggest that MTX and MPA exposure of transduced T cells does not overtly alter T cell surface phenotype, cytolytic function or cytokine levels.

### MTX/MPA-mediated *in vitro* Selection of huEGFRt/DHFR^FS^/IMPDH2^IY^ Transduced Primary Human T cells

**Figure 4:**
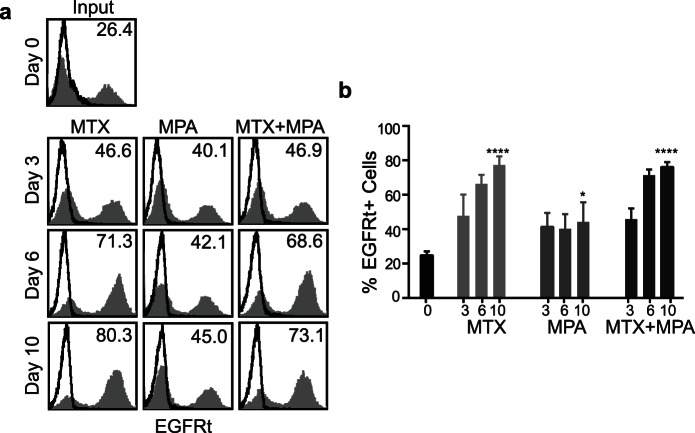
Primary human T cells transduced to express huEGFRt/DHFR^FS^/IMPDH2^IY^ can be selected *in vitro* with MTX and MPA. (**a**), Representative flow cytometric evaluation of EGFRt transgene expression (grey histograms) on transduced T cells over 10 days of culture in 0.1 µM MTX, 1 µM MPA, or 0.05 µM MTX +0.75 µM MPA. Percentage of positive cells above control staining (open histograms) is indicated in each histogram. (**b**), Graphical depiction of the percentages of EGFRt^+^ cells shown in (a). Equal numbers of three different gene-modified T cell lines were each plated in a 6-well plate at the indicated drug concentrations. The data represent means ± S.D. There was a significant difference between the cells on D0 vs. D10 at either 0.1 µM MTX, 1 µM MPA, or 0.05 µM MTX +0.75 µM MPA (****, p≤0.0001; *, p≤0.05).

To determine to what degree the gene modified T cells could be selected *in vitro*, transduced primary human T cells were evaluated following short-term culture with either MTX alone, MPA alone or dual drug-selection with MTX and MPA. For these studies we used huEGFRt/DHFR^FS^/IMPDH2^IY^ T_CM_ that were 24.8±2.3% transgene positive based on EGFRt staining (**Fig. 4**). Cells were then cultured for 10 days in 0.1 µM MTX, 1 µM MPA, or 0.05 µM MTX +0.75 µM MPA. Comparison of huEGFRt expression in 0.1 µM MTX revealed a 3.1±0.3-fold DHFR^FS^-mediated selection from a baseline of 24.8±2.3% to 77.3±5.0% at day 10. (**Fig. 4**). Transduced T cells cultured in MPA were enriched to 44.2±9.2% (a 1.8±0.4-fold selection), while treatment with MTX and MPA enriched the transduced T cells to 76.1±2.8% (a 3.0±0.3-fold selection). Taken together, these findings demonstrate that both DHFR^FS^ and IMPDH2^IY^ can confer drug-mediated selection of T cells in short-term (10-day) *in vitro* cultures, with that mediated by DHFR^FS^ in the presence of MTX apparently being more robust that that mediated by IMPDH2^IY^ in MPA.

### Development of a Non-toxic Drug Regimen for *in vivo* Selection of T cells

**Figure 5:**
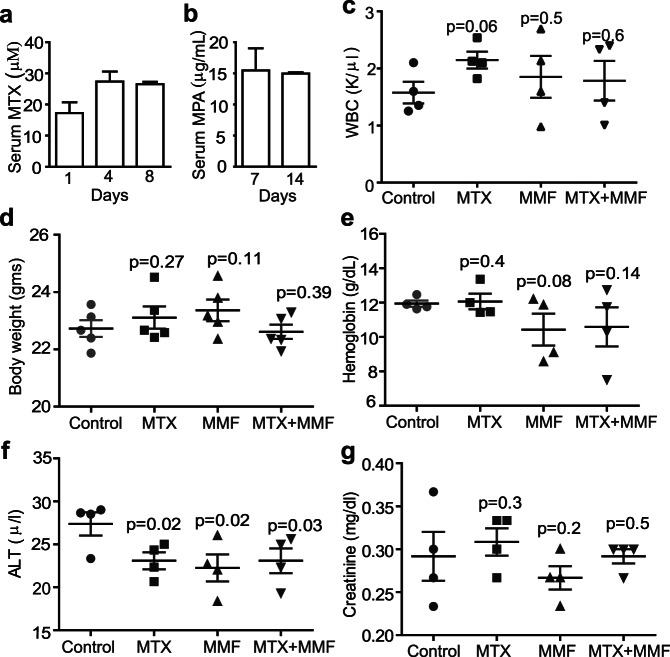
Establish non-toxic MTX and MMF dose regimens for *in vivo* selection. 6–8 week old NSG mice (n = 6) were (**a**), administered MTX by i.p. injection at 25 mg/kg/day twice a week the first week (days 1 and 4) and only once the second week (day 8), and/or (**b**), fed 0.563% MMF medicated feed for 2 weeks. Serum MTX and MPA levels in each case were analyzed by HPLC. Drug toxicity was then monitored by measuring (**c**), white blood cell counts, (**d**), body weight, (**e**), Hemoglobin, (**f**), ALT, and (**g**), creatinine levels. (c–g), Mean levels of each measurement (± S.D) after the last treatment (i.e., at day 14) are depicted. There was no significant difference between control and treatment mice (p≥0.05). Data were evaluated between control and treatment mice using an unpaired, two-tailed Student’s t - test.

To assess the potential of DHFR^FS^ and IMPDH2^IY^ to allow for *in vivo* selection of gene-modified T cells, we first optimized the dosage regimens of MTX and MMF (i.e., the prodrug of MPA) that could be tolerated by NSG mice without deleterious effects. Intraperitoneal administration of MTX 3 times over a 2-week period at 25 mg/kg body weight was found to result in average MTX serum concentrations of 17.3±6.0 µM, 27.4±5.5 µM, and 26.6±1.3 µM when blood was harvested 30 minutes post i.p. injection on days 1, 4, and 8 respectively (**Fig. 5a**). For MMF, mice received medicated feed (0.563% MMF) for two consecutive weeks (**Fig. 5b**) in order to maintain more continuous active serum MPA levels, since MPA is known to convert over time to the inactive metabolite glucuronide via uridine 5′ diphosphoglucuronyltransferase [26]. Serum concentrations of MPA achieved with this delivery regimen were 15.5±6.2 µg/ml at day 7, 15.0±0.35 µg/ml at day 14, and then undetectable 7 days after the medicated feed was withdrawn (day 21). Pilot experiments established that these MTX and MMF dosing regimens were sufficient for controlling the proliferation of a murine lymphoblast cell line (CTLL2) in NSG mice (data not shown). Importantly, we found that these drug regimens were well tolerated by mice. There was no significant decrease or increase in white blood cell counts (**Fig. 5c**), mouse bodyweight (**Fig. 5d**), hemoglobin (**Fig. 5e**), alanine aminotransferase (ALT) as a measure of liver function (**Fig. 5f**), or creatinine levels as a measure of kidney function (**Fig. 5g**) (p≥0.05) compared to control mice. Collectively, these findings suggested that these drug dose concentrations are of minimal of toxicity and enabled the assessment of our DHFR^FS^/IMPDH2^IY^ platform for *in vivo* selection of transduced primary human T cells in NSG mice.

### *In vivo* Selection of Primary Human T cells Transduced with huEGFRt/DHFR^FS^/IMPDH2^IY^

**Figure 6:**
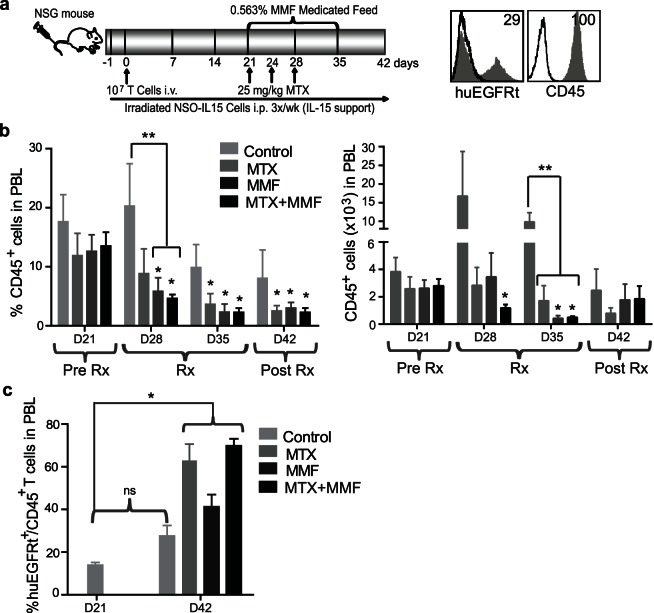
Transduced T cells that express DHFR^FS^/IMPDH2^IY^ exhibit an *in vivo* engraftment advantage upon treatment with MTX and MMF. (**a**), Model system for testing the *in vivo* selection of transduced T cells using MTX, MMF and a combination of both. Depicted is the CD45 cell surface expression (filled grey histogram) and EGFRt expression (filled grey histogram) of transduced T cells prior to adoptive transfer analyzed by flow cytometry. Percentage of positive cells above isotype control staining (open histogram) is indicated in each histogram. 6 to 8 week old NSG (n = 6) mice were injected with 10^7^ T-EGFRt (29% EGFRt^+^) cells i.v. (Day 0). Drugs were administered 21 days post engraftment and analyzed for CD45^+^ cells in peripheral blood. Experimental groups were administered either MTX i.p. 2x in week 1 and 1x in week 2 (25 mg/kg), 0.563% MMF feed for 2 weeks, or a combination of both drugs (i.e., from D21 to D35). (**b**), The average percentage (left) or number (right) of CD45^+^ peripheral blood cells on day 21 before the initiation of treatment, and 7 (D28), 14 (D35), and 21 (D42) days during and after treatment are shown. *P-*value ≤0.05 using unpaired, two-tailed Student’s t-tests as compared to that in D21 (*) or as compared to the Control in the same day (**). (**c**), Percentage of EGFRt^+^ cells in the CD45^+^ population on day 21 before the initiation of treatment and one week after treatment (42) are shown. *, p≤0.0006 when each group was compared the control on D21. There was no significant difference between control mice on D21 and D42 (ns). Mean percentage of EGFRt ± S.E.M. of 6 mice are depicted.

To examine the utility of the DHFR^FS^/MTX and IMPDH2^IY^/MPA selection platforms for selecting genetically engineered T cells *in vivo*, we used a human IL-15 reconstituted NSG mouse model [24] to engraft a mixed population of transduced T cells (29% EGFRt positive) (**Fig. 6a**). The engrafted human cells in mouse peripheral blood were tracked by flow cytometric analysis of human CD45 and EGFRt. Prior to drug treatment, the mean percentage of CD45 cells in the peripheral blood of all groups of mice was 12.6±2.8 at day 21. MTX and/or MPA were then administered to the mice for two weeks (**Fig. 6a**), and seven days after the drug dosing regimens were completed (day 42), the percentage of engrafted CD45^+^ cells significantly decreased to 2.1±0.9 (p = 0.015) for MTX, 3.0±0.9 (p = 0.005) for MMF, and 1.9±0.7 (p = 0.0004) after combined MTX and MMF administration. This is in contrast to that seen in the control group (6.7±4.1; p = 0.133), which exhibited CD45^+^ T cell engraftment that was approximately 2–3 fold (p≥0.05) higher than that observed in the drug-administered mice (**Fig. 6b**). Numbers of engrafted CD45^+^ cells were also significantly decreased compared to controls at the end of the dosing regimen (day 35), i.e., from 10.3×10^3^±2.4×10^3^ for the controls, to 1.7×10^3^±1.1×10^3^ (p = 0.01) for MTX, 0.4×10^3^±0.19×10^3^ (p = 0.008) for MMF, and 0.49×10^3^±0.08×10^3^ (p = 0.009) for combined MTX and MMF.

To evaluate the impact of drug administration on the engraftment of huEGFRt/DHFR^FS^/IMPDH2^IY^ T_CM_ versus unengineered T cells, the mean percentages of EGFRt^+^ expressing T cells within the CD45^+^ human T cell populations were compared for each treatment group. The EGFRt^+^ T cells detected in the peripheral blood before initiation of treatment were comparable for all groups at 15.0±8.4% on day 21 after adoptive transfer of transduced T cells. At day 42, 3 weeks after the initiation of drug administration, the mean percentage of EGFRt^+^ expressing T cells was increased from this baseline to 63±7.7% (p = 0.0002) in mice treated with MTX, and 70±3.2% (p<0.0001) in combination MTX+MMF-treated mice, but only to 41±5.7% (p = 0.0006) in mice treated with MMF alone. Each drug dosing regimen exhibited higher percentages of EGFRt^+^ cells than that seen in control animals, which was 27.6±4.9% (p = 0.26 vs. control animals on D21) (**Fig. 6c**). The increased percentages of transgene expressing T cells within the human CD45^+^ populations in mouse peripheral blood indicated that the huEGFRt/DHFR^FS^/IMPDH2^IY^-expressing T cells were able to resist lymphotoxic drug concentrations and persist *in vivo* with a selection advantage over the non-transduced T cells.

## Discussion

In human gene therapy, the selection of genetically modified cells is a methodological challenge when potency and safety are linked to the purity of *ex vivo* manufactured cell products. Selection platforms based on non-human drug resistance genes often lead to immune-based rejection of the therapeutic product [2]–[6], whereas those employing immunomagnetic or sorting methods are technically cumbersome and limited by expense and availability of clinical-grade reagents. Alternately, cell selection can be achieved by chemical means on the basis of human enzymes that confer resistance to cytotoxic selection drugs. It has been previously shown that MTX resistant hDHFR variants have an excellent potential as selectable markers for gene transfer in hematopoietic stem cells (HSCs), resulting in improved protection of HSCs from MTX cytotoxicity [27]. Furthermore, *ex vivo* selection studies on bone marrow cells transduced with various hDHFR variants demonstrated that the DHFR^FS^ variant displayed decreased MTX affinity [28], with retention of enzymatic activity to allow for cell proliferation [29]. Nonetheless, *ex vivo* MTX selection of murine or human hematopoietic cells with DHFR^FS^ did not lead to an increased reconstitution of transduced cells in myelo-ablated animal recipients [30], [31], which might be due to the time required for efficient selection exceeded the *ex vivo* life span of HSCs. Similarly, previous studies have also shown that IMPDH2^IY^ expressing lymphocytes can be selected *in vitro* using MPA [8]. Here, we now demonstrate the utility of both human mutant enzymes DHFR^FS^ and IMPDH2^IY^ as a strategy to enrich transduced T cells *in vitro* and *in vivo* for adoptive T cell therapy.

Our results demonstrate that DHFR^FS^/IMPDH2^IY^ co-expression renders lentivirally transduced primary human CD45RO^+^CD62L^+^ central memory T cells resistant to lymphotoxic concentrations of MTX and MPA (up to 0.1 µM and 1.0 µM, respectively) *in vitro*. Further, our data provide evidence that MTX, MMF and dual drug selection efficiently enriches gene-modified T cells *in vitro* within a short expansion time (12 days) (DHFR^FS^/MTX 11.4±2.1-fold; IMPDH2^IY^/MMF 3±0.9-fold; and MTX/MMF dual drug selection 5.4±1.0-fold), without compromising the cytolytic or cytokine production capabilities of the gene-modified T cells. Our recent *in vitro* work with primary human T cells lentitransduced to coordinately express just the DHFR^FS^ with huEGFRt as well as a CD19-specific chimeric receptor revealed a similar DHFR^FS^/MTX mediated selection and expansion profile [32]. Now, based on the *in vitro* analysis presented here, we speculate that the dual expression of DHFR^FS^/IMPDH2^IY^ by drug selected T cells will render adoptively transferred cells resistant to clinically relevant serum concentrations of both MTX (0.1–1 µM ) [33] and MMF (1–10 µM) [34], [35].

We also demonstrate the survival advantage and enrichment of DHFR^FS^/IMPDH2^IY^ engineered T cells upon drug selection *in vivo*. After establishing MTX and MMF dosing regimens that are well tolerated by NSG mice, we were able to observe the enrichment of huEGFRt^+^DHFR^FS+^IMPDH2^IY+^ T cells following either administration of MTX alone (4.4 -fold), MMF alone (2.9-fold), or combined MTX and MMF (4.9-fold).

We originally incorporated two drug-resistance transgenes in our selection approach with the idea that it would reduce the necessity for utilizing large doses of either drug individually to drive selection of gene-modified cells. However, the *in vivo* results suggest that the DHFR^FS^/MTX strategy alone may be sufficient for potent selection, in that it appears more robust than the IMPDH2^IY^/MMF strategy alone, and is comparable to dual selection with DHFR^FS^/MTX and IMPDH2^IY^/MMF together. This correlates with the observed *in vitro* expansion of DHFR^FS^/IMPDH2^IY^-transduced T cells, which was less robust in MPA compared to that seen in MTX. We presume that MMF inhibition of the guanosine nucleotide pools, which is notably difficult for cells to recover from compared to inhibition of other nucleotides [36]–[38], is responsible for the reduced selection-potential of IMPDH2^IY^/MMF over that of DHFR^FS^/MTX in our T cells.

Another appealing attribute of the DHFR^FS^/IMPDH2^IY^-mediated selection system includes the ability of MTX and MMF administration to induce lymphopenia while reinforcing transgene expression, with the potential outcome of selective homeostatic cytokine-driven engraftment of transferred gene-modified T cells over that of endogenous T cells. Further, not only would these lymphotoxic drugs favor the proliferation of the transduced cells, but they might also substantially prevent immune responses toward transgene-encoded proteins by suppressing immunological responses. Thus, this platform may be particularly useful in allogeneic applications of adoptive T cell therapy. Lastly, MTX and MMF are used clinically against a variety of hematologic malignancies and may thus provide additive or synergistic anti-tumor effects when used in the adoptive T cell therapy setting.

In summary, this is the first study to demonstrate the feasibility of DHFR^FS^/IMPDH2^IY^ mediated *in vitro* and *in vivo* enrichment of primary human T cells with MTX and MPA and to facilitate selective engraftment of gene-modified cells following transfer *in vivo.* We are currently evaluating additional studies in animal models to assess the DHFR^FS^/MTX-mediated selection of therapeutic (i.e., chimeric antigen receptor expressing) primary human T cells *in vivo* [32].

## Materials and Methods

### Cell Lines and Maintenance

The human peripheral blood mononuclear cells (PBMCs) were isolated as described [24] from heparinized peripheral blood obtained from discard kits containing residual blood components of healthy donors that had undergone apheresis at the City of Hope National Medical Center (COHNMC). Because this was de-identified discard blood material, informed consent was waived with the approval of the COHNMC Internal Review Board (IRB protocol #09025), and the COHNMC Office of Human Subjects Protection. T_CM_-derived T cell isolation, stimulation, expansion (Rapid expansion method; REM) and lentiviral-mediated transduction were then done as previously described by our laboratory [24].

EBV-transformed lymphoblastoid cell lines (LCL) and LCL that expressed OKT3 (LCL-OKT3) cells [24] were cultured in RPMI 1640 (Irvine Scientific, Santa Ana, CA) supplemented with 10% heat-inactivated fetal calf serum (FCS, Hyclone, Logan, UT) 2 mM L-glutamine (Irvine Scientific), and 25 mM HEPES (Irvine Scientific).

Mouse myeloma cells secreting human homeostatic IL-15 cytokine (NSO-IL15) cells were generated by transfecting the NSO cells with GFP-IMPDH2^IY^-2A-IL15_pcDNA3.1. The transfected NSO cells were cultured and expanded as previously described by our laboratory [24]. The transfected cells were selected under MPA, analyzed for the GFP expression by flow cytometry and human IL-15 secretion by cytometric bead array (Bio-Rad Laboratories, Hercules, CA, USA) [24].

### Lentiviral Vector

For gene-modification of T cells (i.e., the T_CM_-derived T cells as described in *Cell lines and maintenance* section above), the huEGFRt-T2A-DHFR^FS^-T2A-IMPDH2^IY^_epHIV7 vector was made and tested for resistance to both MMF and MTX. We generated double mutant DHFR^FS^ and IMPDH2^IY^ transgenes by site-directed mutagenesis, inserting two point mutations in wild type human DHFR and IMPDH2. The DHFR^FS^ [16] and IMPDH2^IY^ [8] selection genes along with a truncated EGFR [22] marker gene (EGFRt expression reflected drug resistance in transduced cells) was incorporated into a lentiviral vector epHIV_7 under transcriptional control of the human elongating factor 1-α (EF-1α) promoter. In the vector, the EGFRt, DHFR^FS^ and IMPDH2^IY^ coding sequences were separated by the ribosomal skip T2A [23] sequence for translation of the three proteins from one transcribed message. The EGFRt gene was placed upstream of DHFR^FS^, and the IMPDH2^IY^ gene(s) was placed downstream of the T2A sequence in a plasmid (**Fig. 1c**). The proper assembly of the genes was verified by DNA sequence analysis.

EGFRt-T2A-DHFR^FS^-T2A-IMPDH2^IY^_epHIV7 lentiviral vector was produced by transient transfection of 293T cells with four plasmids (pCgp, pCMV-G, pCMV-Rev2 and vector construct) using the CaPO_4_ method. The supernatant containing secreted lentiviral vector was harvested 24, 48 and 72 hours after transfection and ultra-concentrated following polyethylene glycol precipitation and titered on H9 cells for EGFRt expression by FACS analysis. Replication-competent lentivirus (RCL) assays were performed using the HIV-1 p24 antigen ELISA kit (Perkin Elmer Life Sciences, California) to detect p24 viral antigen. RCL negative viral supernatant was released for use.

### Flow Cytometry

Cell surface phenotypes of transduced and non-transduced purified CD62L^+^CD45RO^+^ healthy donor peripheral blood T cells were analyzed with fluorochrome-conjugated streptavidin and antibodies specific for CD4, CD8, CD62L, CD45 RA, CD45RO, CD45, CCR7, TCRαβ, CD127, and CD28 and isotype matched controls (BD Biosciences) following manufacture’s recommendations. The generation of the biotinylated-cetuximab has been previously described [22]. The percentage of immunofluorescent cells were analyzed by a FACScalibur system (BD Biosciences), and the percentage of cells in a region of analysis were calculated using FCS Express V3 (De Novo Software, CA, USA).

### *In vitro* Selection of Gene-modified T cells

The concentration for *in vitro* selection of gene-modified T cells in the presence of MTX (Parenta Pharmaceuticals, Yardley, PA), MPA (Sigma-Aldrich, St. Louis, MO) and a combination of MTX and MPA was determined by dose-response curve. The selection experiment was initiated on day 8 of REM stimulation cycle by plating 0.8×10^6^ transduced primary human T cells in 24-well tissue culture plates and culturing for 12 days with MTX, MPA or a combination of both drugs. Drug-selected or treated T cells were analyzed for transgene selection (EGFRt^+^) efficiency by flow cytometry. Transduced T cells with or w/o cetuximab were immunomagnetically selected for EGFRt expression [22] and were evaluated for their resistance to the indicated concentrations of MTX and MPA. The total viable cell number and percentage of viable cells were enumerated by Guava Personal Cell Analysis (Guava Technologies-Millipore, Hayward, CA) as per the manufacturer’s instructions. The working concentrations of MTX and MPA were diluted in PBS.

### Chromium-release Assays

The cytolytic activity of T cells was determined by a 4-hour chromium-release assay (CRA) previously described [24] using the indicated effector/target cell ratios.

### Measurement of Cytokine Production

T cells (5×10^4^) were co-cultured with 5×10^4^ LCL-OKT3, LCL, or K562 cells in triplicate wells of a 96-well tissue culture plate overnight. The supernatants were then analyzed for the secreted cytokines by cytometric bead array using a bioplex human cytokine Panel (Bio-Rad Laboratories, Hercules, CA, USA), according to the manufacturer’s instructions.

### Determination of Plasma MTX/MPA Levels and Effect on NSG Mice

NOD-scid IL2γc −/− (NSG) mice (Jackson Laboratory) were housed and maintained in a breeding colony in individually ventilated conditions under pathogen-free conditions at the COH animal resources center. All procedures were reviewed and approved by the COH Institute Animal Care and Use Committee (#08004). All mice were on a standard rodent diet.

To determine plasma MTX/MPA levels in NOD-*scid* IL2Rγ^null^ (NSG) mice, MTX (Parenta Pharmaceuticals, Yardley, PA) was administered as an intraperitoneal (i.p.) injection at 25 mg/kg/day twice a week the first week, and only once the second week. MMF (Sandoz Ltd, India) was administered in 0.563% medicated feed pellets (from pulverized tablets) for 2 weeks. MMF medicated pellets were manufactured (TestDiet, Richmond, IN) with the addition of 5.625 mg MMF per 1 gm of standard rodent diet. The blood samples were collected for the determination of plasma levels of MTX in heparinized microcapillary tubes from the retro-orbital venous plexus 30 minutes after each i.p. administration of 25 mg MTX/kg (this was based on t1/2 [39]), or 7 days after initiation of 0.563% MMF feed. Measured plasma levels of MTX were performed at the Molecular Pharmacology laboratory at COH and plasma levels of MPA were determined at the Transplant Pharmacokinetic Laboratory Dumont (UCLA Liver Transplant Center) by standard high-performance liquid chromatography. Further effects of these drugs were monitored by measuring animal body weights and observing general health condition on a weekly basis. Complete blood counts were determined (HEMAVET 950FS) and serum chemistry was analyzed (Charles River Research Diagnostic Services, Wilmington, MA) before, during and after drug administration.

### *In vivo* T cell Engraftment

Six- to ten-week old NSG mice were injected intravenously on day 0 with 10^7^ T cells. The systemic supply of human IL-15 was provided by injecting the irradiated (8000 cGy) 2×10^7^ NS0-IL15 cells i.p. on Monday, Wednesday and Friday starting on day -1 to support human T cell expansion *in vivo*. Mouse peripheral blood was collected by retro-orbital bleeding, and analyzed by flow cytometry to monitor human T cell engraftment. Flow cytometric analysis included the use of CountBright™ Absolute Counting Beads (Molecular Probes, Eugene, OR) for normalization of samples according to the manufacturer’s directions. In the case of selection studies, the transferred T cells were allowed to engraft for 21 days. MTX was then administered i.p. at a 25 mg/kg/day twice a week the first week and only once in the second week; MMF was administered as 0.563% medicate feed pellets (from pulverized tablets) for 2 consecutive weeks starting from day 21.

### Statistical Analysis

Data are expressed as mean ± S.D. To evaluate the differences between non-transduced and transduced cells in terms of total percentage of viable cells and fold expansion were analyzed using an unpaired, two-tailed Student’s t-test. As well, to test for differences between treatment and control mice, data were evaluated using an unpaired, two-tailed Student’s t - test in Graph Pad Prism5. Mean percentage of EGFRt ± S.E.M. of 6 mice are depicted. Differences were considered statistically significant if p values were less than 0.05.

## Supporting Information

Figure S1 (EPS)
